# Urinary APE1/Ref-1: A Potential Bladder Cancer Biomarker

**DOI:** 10.1155/2016/7276502

**Published:** 2016-01-21

**Authors:** Sunga Choi, Ju Hyun Shin, Yu Ran Lee, Hee Kyoung Joo, Ki Hak Song, Yong Gil Na, Seok Jong Chang, Jae Sung Lim, Byeong Hwa Jeon

**Affiliations:** ^1^Research Institute of Medical Sciences, Department of Physiology, School of Medicine, Chungnam National University, Daejeon 35015, Republic of Korea; ^2^Department of Urology, Chungnam National University Hospital, Daejeon 35015, Republic of Korea; ^3^Department of Physiology, College of Medicine, Seonam University, Namwon 55724, Republic of Korea

## Abstract

Bladder cancer (BCa) is one of the most common urothelial cancers with still noticeable incidence rate. Early detection of BCa is highly correlated with successful therapeutic outcomes. We previously showed that apurinic/apyrimidinic endonuclease 1/redox factor-1 (APE1/Ref-1) was expressed at an increased level in the serum of BCa patients when compared to the level in healthy controls. In this study, we investigated whether urinary APE1/Ref-1 was also elevated in patients with BCa. In this case-control study, voided urine was collected from 277 subjects including 169 BCa patients and 108 non-BCa controls. Urinary APE1/Ref-1 level was assessed by enzyme-linked immunosorbent assay (ELISA). APE1/Ref-1 levels were significantly elevated in BCa patients relative to levels in non-BCa controls and were correlated with tumor grade and stage. Urinary APE1/Ref-1 levels were also higher in patients with recurrence history of BCa. The receiver operating characteristics (ROC) curve of APE1/Ref-1 showed an area under the curve of 0.83, indicating the reliability and validity of this biomarker. The optimal combination of sensitivity and specificity was determined to be 82% and 80% at a cut-off value of 0.376 ng/100 *μ*L for detection of APE1/Ref-1 in urine. In conclusion, urinary APE1/Ref-1 levels measured from noninvasively obtained body fluids would be clinically applicable for diagnosis of BCa.

## 1. Introduction

Bladder cancer (BCa) is the second most common of all genitourinary malignancies in the United States [1] and Korea [2]. Most individuals with BCa who are diagnosed early, show no muscle invasion, and have superficial urothelial carcinoma can expect a 5-year survival rate of more than 90% [3]. If the BCa is invasive, however, with tumor cells spreading beyond the bladder to the surrounding tissue or to nearby lymph nodes, or organs, signs of late stage BCa, the 5-year survival rate drops sharply. Therefore, early intervention can dramatically increase the probability of a BCa patient's survival. Patients with noninvasive BCa frequently show a high rate of recurrence and progression of the disease within 2 years of transurethral resection [4], and continuous follow-up testing is required. Several studies have focused on the development of tools for the diagnosis and prognosis of BCa using urinary biomarkers [5–9].

In our previous report, we proposed a new BCa diagnostic protein, apurinic/apyrimidinic endonuclease 1/redox factor-1 (APE1/Ref-1) in serum [10]. APE1/Ref-1 protein was originally identified as a multifunctional protein involved in DNA repair and redox signaling. APE1/Ref-1 levels were found to be elevated with dysregulated cellular proliferation, as is typically seen in cancers [10–12]. APE1/Ref-1 is mainly localized in the nucleus and shows dynamic shuttling between the nucleus and cytoplasm in response to various stress stimuli. Furthermore, extracellular secretion of APE1/Ref-1 into the circulation suggests this protein could be used as a serologic biomarker [13, 14]. APE1/Ref-1 secretion from cells is also supported by the presence of autoantibody against APE1/Ref-1 in the blood of patients with lung cancer [15] and systemic lupus erythematosus [16].

The clinical need for specific and sensitive urothelial tumor diagnostics remains as urgent issue. The ideal diagnostics would measure the level of a BCa protein biomarker in a single step with a noninvasive sampling method. The protein components of urine are qualitatively similar to those of blood although they are more diluted [17]. A diagnostic based on patient urine has some advantages as urine is available in large quantities and can be obtained by noninvasive means for repeated measurements, continuous surveillance, or establishing of assay reproducibility.

In this study, we compared the urinary APE1/Ref-1 levels of patients with BCa and healthy subjects. Using an existing, quantitative APE1/Ref-1 serum assay that was modified for use with urine sample, we aimed to quantify the level of APE1/Ref-1 in urine samples from patients with results from cystoscopic examination of the bladder to establish a new, noninvasive urinary BCa biomarker.

## 2. Materials and Methods

### 2.1. Patients and Urine Samples

Urine samples were obtained from 277 patients. All analyses were performed within 6 months of collection. The study subjects were classified as either noncancer controls (*n* = 108), individuals with no evidence of malignancy, or patients with bladder tumors (*n* = 169). Control patients were diagnosed as having nonmalignant urological diseases including benign prostate hyperplasia (*n* = 37), neurogenic bladder (*n* = 23), urolithiasis (*n* = 21), stress urinary incontinence (*n* = 19), and urethral stricture (*n* = 8). Tumors were staged according to the 2011 tumor-node-metastasis (TNM) classification [18] and graded according to the World Health Organization (WHO) system [19] by two pathologists that were blind to this study. In the BCa group, postoperative histological confirmation of urothelial cell carcinoma, including grade and stage, was recorded. All patients' clinical pathological data, including prior medical history and treatment, recurrence, and clinical stage, were retrieved from electronic medical records (Table 1). Voided urine samples were obtained from patients with BCa before cystoscopy and then frozen in liquid nitrogen and stored at −70°C until use. All samples were obtained from the archives of the Department of Urology, Chungnam National University, from 2011 to 2012. The collection and analysis of all samples were approved by the Chungnam National University Hospital institutional review board, and informed consent was obtained from all study subjects.

### 2.2. Urine Sample Preparation

Each urine sample was thawed and sedimented by centrifugation (10,000 ×g for 10 min) to remove impurities. The supernatant was carefully collected and prepared without an additional protein enrichment step.

### 2.3. Measurement of Urinary APE1/Ref-1

The level of APE1/Ref-1 in each urine sample was quantitatively analyzed using a sandwich enzyme-linked immunosorbent assay (ELISA) as described previously [10], with some modification. Briefly, 96-microwell plates (Thermo Fisher Scientific, Waltham, MA, USA) were precoated overnight with 100 *μ*L of a 1 : 5000 dilution of a rabbit anti-APE1/Ref-1 antibody (Abcam, Cambridge, UK) in coating buffer (0.5 M carbonate buffer, pH 9.6) in each well. Plates were washed five times with phosphate buffered saline (PBS) containing 0.05% Tween 20 (PBS-T) between all incubation steps. After blocking with blocking buffer (5% bovine serum albumin in PBS-T) at room temperature for 2 h, plates were washed five times with PBS-T. Urine sample, standards, and blank (100 *μ*L/well) were added to the wells. Plates were incubated at 37°C for 90 min and then washed five times with PBS-T to ensure the removal of remnants that could affect antibody recognition. This was followed by the addition of 100 *μ*L of a 1 : 5000 dilution of a mouse anti-APE1/Ref-1 antibody (Abcam, Cambridge, UK) and further incubation at room temperature for 2 h. The plates were then washed seven times with PBS-T, and 100 *μ*L of horseradish peroxidase-conjugated anti-mouse antibody (1 : 5000) was added; the plate was incubated at room temperature for 30 min. After washing, 100 *μ*L of freshly prepared tetramethyl benzidine substrate was added to the wells. The color development reaction was stopped by adding 100 *μ*L of 2.5 M H_2_SO_4_, and the absorbance was measured at 450 nm with an automatic microtiter plate reader (Sunrise Xfluor4; Tecan Systems, Inc., San Jose, CA, USA). Each sample was assayed in duplicate, and mean values were determined. A 6-point standard curve was established using purified recombinant human APE1/Ref-1 (rh APE1/Ref-1) [10]. The protein (1 *μ*g/mL) was serially diluted (5-fold) in diluent and used at 0.0064–4 ng/100 *μ*L.

### 2.4. Statistical Analysis

Data were expressed as the mean ± standard error of the mean (SEM). A repeated-measures ANOVA was used to compare more than three variables, while Student's* t*-test was used to evaluate differences between two variables. Receiver operating characteristic (ROC) curves were generated by plotting the sensitivity value against the false-positive rate (1 − specificity). We assessed the predictive value of APE1/Ref-1 for BCa by calculating the area under the curve (AUC), and we estimated the optimal cut-off value (Youden index), based on maximum sensitivity and specificity [20]. SPSS version 18.0 for Windows was used to analyze the data. Differences were considered statistically significant if the null hypothesis could be rejected with >95% confidence (*p* < 0.05).

## 3. Results

### 3.1. Urine Levels of Human APE1/Ref-1 in Patients with Bladder Cancer

**Figure 1:**
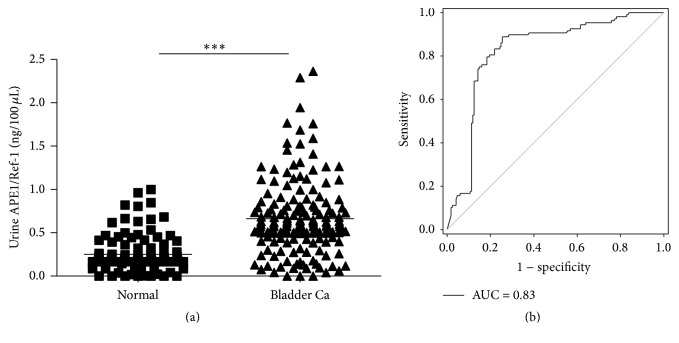
Urinary APE1/Ref-1 levels are elevated in bladder cancer. (a) Urine APE1/Ref-1 levels were measured using an enzyme-linked immunosorbent assay (ELISA). The results are presented as a scatter plot. Each dot represents one patient (*n* = 108 for noncancer controls; *n* = 169 for bladder cancer patients). (b) Receiver operating characteristics (ROC) curves of APE1/Ref-1 in bladder cancer detection. The area under the curve (AUC) for the detection of all cancers by APE1/Ref-1 was 0.826.

To determine whether urine APE1/Ref-1 levels are elevated in patients with BCa, we used a sandwich ELISA for human APE1/Ref-1 with rh APE1/Ref-1 protein as a standard. Urine levels of APE1/Ref-1 in patients with BCa were significantly higher than those in the control subjects (0.663 ± 0.032 ng/100 *μ*L (*n* = 169) for BCa patients, 0.250 ± 0.020 ng/100 *μ*L (*n* = 108) for the control group, *p* < 0.01 (Figure 1(a))). ROC analysis of control versus BCa groups (Figure 1(b)) yielded an AUC of 0.83; the ROC curve and corresponding AUC show that urine APE1/Ref-1 as a biomarker has the predictive ability to discriminate between patients with BCa and normal subjects. The optimal cut-off value was set to maximize the sum of sensitivity and specificity. Based on these results, the optimal combination of sensitivity (81.7%) and specificity (79.6%) was obtained at a cut-off value of 0.3765 ng/100 *μ*L (Table 2).

### 3.2. Urine APE1/Ref-1 Levels Are Associated with Bladder Tumor Grade, Stage, and Recurrence

**Figure 2:**
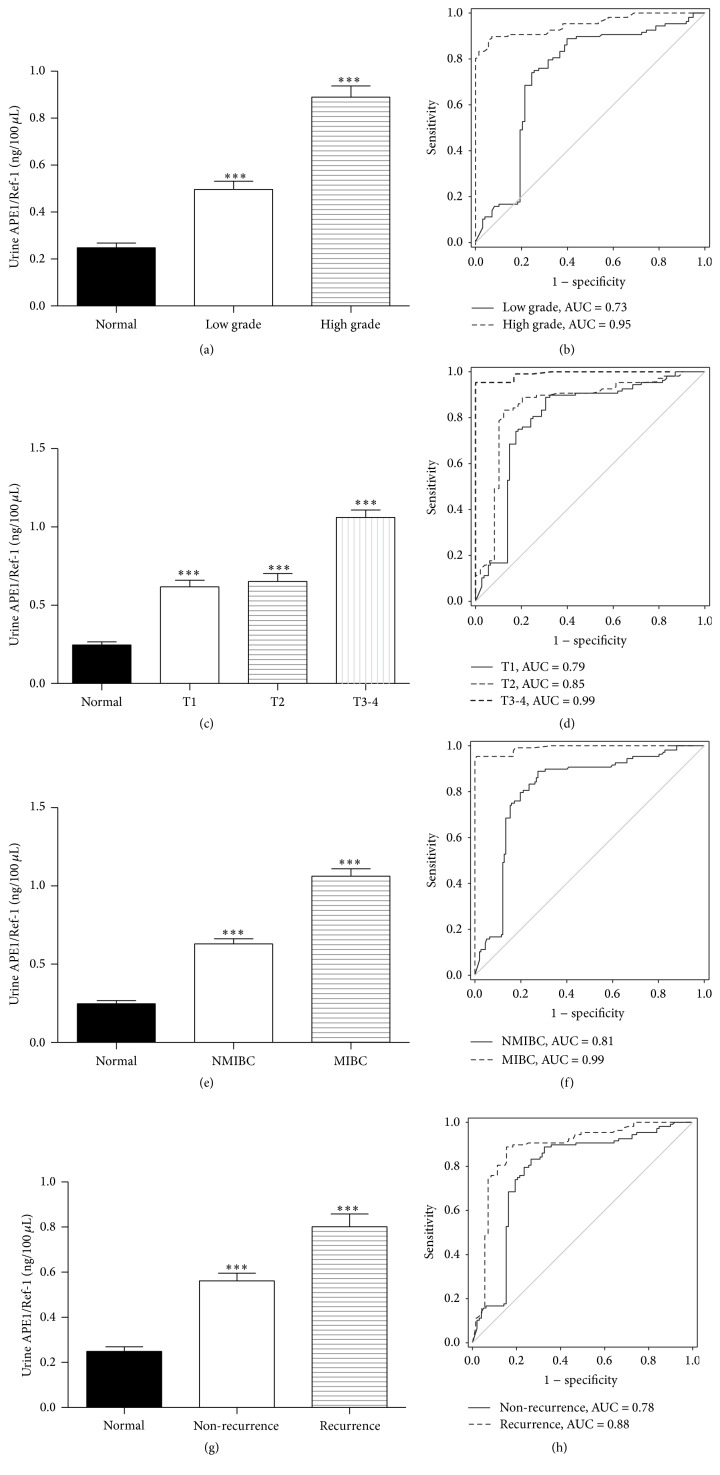
Urinary APE1/Ref-1 levels are associated with bladder tumor grade, stage, muscle invasion, and recurrence. (a) Each bar shows the mean ± standard error of the mean (SEM) (*n* = 98 for low-grade tumors, *n* = 71 for high-grade tumors, and *n* = 108 for noncancer controls). (b) ROC curves for APE1/Ref-1, the determination of different bladder tumor grades. (c) Urinary APE1/Ref-1 levels are elevated in patients with higher stage tumors. Each bar shows the mean ± SEM (*n* = 49 for stage T1, *n* = 10 for stage T2, *n* = 2 for stage T3-T4, and *n* = 108 for noncancer controls). (d) ROC curves for APE1/Ref-1, the determination of different bladder tumor stages. (e) Urinary APE1/Ref-1 levels are higher in patients with muscle-invasive bladder cancer. Each bar shows the mean ± SEM (*n* = 157 for non-muscle-invasive bladder cancer [NMIBC], *n* = 12 for muscle-invasive bladder cancer [MIBC], and *n* = 108 for noncancer controls). (f) ROC curves for APE1/Ref-1, the determination of different of NMIBC and MIBC. (g) APE1/Ref-1 levels are higher in patients with previously recurrent tumors (*n* = 71 for previously recurrent tumors and *n* = 98 for nonrecurrent tumors). (h) ROC curves for APE1/Ref-1, the determination of recurrent bladder tumors.* Bars*: SEM. ^*∗∗∗*^ *p* < 0.01, significantly different from noncancer controls.

The association between levels of APE1/Ref-1 in urine and tumor grade, stage, invasion status, and recurrence were investigated to determine whether APE1/Ref-1 levels are predictive of BCa diagnosis. As shown in Figure 2(a), significant differences in median urinary APE1/Ref-1 levels were found between control (*n* = 108) and low-grade tumor groups (0.498 ± 0.035 ng/100 *μ*L, *n* = 98; *p* < 0.01) and between control and high-grade tumor groups (0.982 ± 0.048 ng/100 *μ*L, *n* = 71; *p* < 0.01). The ROC AUC for low-grade and high-grade BCa were 0.73 and 0.95, respectively (Figure 2(b)). These results suggest that the increased urinary APE1/Ref-1 levels correlate with severity of BCa disease and, therefore, can help guide treatment. We also determined the association of urinary APE1/Ref-1 levels and BCa stage and depth of tumor invasion into the bladder wall. The urinary APE1/Ref-1 levels were significantly elevated in patients with T1 (0.622 ± 0.043 ng/100 *μ*L), T2 (0.657 ± 0.049 ng/100 *μ*L), and T3-T4 (1.065 ± 0.048 ng/100 *μ*L) stage BCa, compared to levels in subjects of the control group (Figure 2(c)), indicating that urinary APE1/Ref-1 level may discriminate between tumor stages. The ROC AUC for T1, T2, and T3-T4 stages were 0.79, 0.85, and 0.99, respectively (Figure 2(d)). In addition, the median urinary APE1/Ref-1 level in patients with non-muscle-invasive BCa (NMIBC; 0.633 ± 0.033 ng/100 *μ*L, *n* = 157) was significantly lower than that in patients with muscle-invasive bladder cancer (MIBC; 1.065 ± 0.048 ng/100 *μ*L, *n* = 12, *p* < 0.01; Figure 2(e)). The ROC AUC for NMIBC and MIBC were 0.81 and 0.99, respectively (Figure 2(f)). Both BCa groups had significantly higher levels than the controls (control versus NMIBC *p* < 0.01; control versus MIBC *p* < 0.01). As shown in Table 2, 71 patients (42%) had history of recurrence in the 169 patients with bladder cancer. Urinary APE1/Ref-1 levels in patients with previously recurrent BCa (0.803 ± 0.057 ng/100 *μ*L) were significantly higher than in patients without recurrence (0.563 ± 0.034 ng/100 *μ*L, *p* < 0.01; Figure 2(g)). In ROC analysis, the AUC for patients without recurrent BCa was 0.78, and the AUC for patients with previously recurrent BCa was 0.88 (Figure 2(h)).

### 3.3. Urine Levels of APE1/Ref-1 in Patients with Hematuria

**Figure 3:**
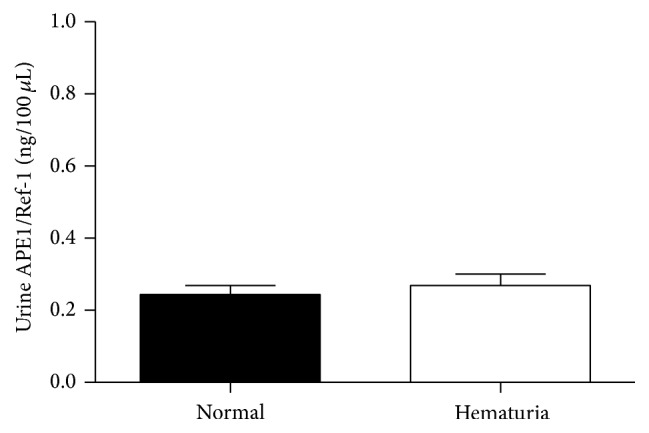
Urine APE1/Ref-1 levels in noncancer controls with hematuria (*n* = 30) did not differ significantly from the levels in noncancer controls without hematuria (*n* = 78).* Bars*: SEM. *p* > 0.5.

False-positive results in the nuclear matrix protein 22 (NMP22) test have been observed in cases with hematuria [21]. Therefore, we investigated the effect of hematuria on urine APE1/Ref-1 levels in human subjects. As shown in Figure 3, urine APE1/Ref-1 levels in controls with microscopic or gross hematuria were not significantly different from levels in controls without hematuria (0.270 ± 0.031 ng/100 *μ*L, controls with hematuria, *n* = 30; 0.243 ± 0.025 ng/100 *μ*L, control without hematuria, *n* = 78, *p* = 0.596). Urine APE1/Ref-1 levels of controls with hematuria (0.270 ± 0.031 ng/mL, *n* = 30) were significantly lower than those of BCa group (0.663 ± 0.032 ng/100 *μ*L, *n* = 169, *p* < 0.01). These results show that urine APE1/Ref-1 levels were not affected by microscopic and gross hematuria.

## 4. Discussion

In the present study, to measure urinary APE1/Ref-1 levels, we modified an APE1/Ref-1 ELISA that was initially developed for the detection of serum APE1/Ref-1 [10]. We obtained an ROC curve by applying results of human urine samples testing and determined a Youden index-based, optimal cut-point for the urinary diagnostic test [20]. A cut-off APE1/Ref-1 value of 0.3765 ng/100 *μ*L resulted in a sensitivity of 81.7% and a specificity of 79.6% of the assay. We also observed that APE1/Ref-1 protein levels were elevated in BCa patients, even those with low-grade tumors, compared with the levels in normal controls. Furthermore, the increased urinary APE1/Ref-1 levels were associated with BCa severity, suggesting that this protein could be used as a reliable marker for BCa diagnosis.

We previously reported serum APE1/Ref-1 as a BCa biomarker [10]. However, to date, there are still no blood-based biomarker tests for use in diagnosis or surveillance of BCa in clinical practice although markers in blood are useful in determining prognosis and informing therapeutic decisions. However, particularly for monitoring bladder diseases, biomarkers in urine have distinct advantages, including larger sample quantities, noninvasive sampling for repeated measurements, and less concern about pathogens transmission than with a blood sample. In general, the proteins in urine originate from glomerular filtration of blood, excretion from epithelial cells in the urinary tract, sloughing of epithelial cells and casts, and formation of urinary exosomes [22]. Changes in urine protein components and concentrations may directly indicate dysfunction of urothelial cells that line the uropoetic organs within the bladder. Therefore, measurement of urinary APE1/Ref-1 levels for BCa monitoring is a rational strategy. However, further studies are needed to determine the origin of urinary APE1/Ref-1 in the blood-stream or bladder itself, the mechanism of excretion into urine, and the meaning of the blood to urine APE1/Ref-1 ratio; urinary APE1/Ref-1 can be established as a new BCa biomarker. However, the results from our urinary APE1/Ref-1 analysis demonstrate an acceptable sensitivity, specificity, and reliability as a BCa diagnostic.

APE1/Ref-1 has been implicated in the development and progression of various cancers [10–12, 23]. Genetic variants of APE1/Ref-1 have been also studied to determine the relationship between specific polymorphisms and cancer susceptibility. Because of its frequency, the most well studied genetic variant is Asp148Glu; D148E [24]. Interestingly, APE1/Ref-1 has been shown to exhibit an atypical subcellular distribution pattern and cytoplasmic localization in many cancer types [25]. Based on our finding and those of previous reports, we propose that the high frequency and cytoplasmic localization of the genetic variant D148E APE1/Ref-1 in BCa cells are associated with the existence of this protein in urine.

In this study, we used frozen urine samples. To avoid alteration of the protein composition during frozen storage, we minimized the number of freeze-thaw cycles (1-2) by preparing small aliquot of each urine sample. Further, precipitates containing cellular debris, which may bias test results, were removed from the urine before storage. However, it is possible that freshly voided urine samples will yield different results. The cellular contribution of proteins derived from bladder or renal tissues or squamous epithelial cells from the urethra or external genitalia also cannot be ruled out.

In our analysis, we carefully considered the effects of hematuria, urinary hemoglobin levels when APE1/Ref-1 levels in urine were monitored by ELISA, because many people with hematuria have been evaluated for BCa, and an APE1/Ref-1 ELISA needs to be performed with accuracy despite the presence of hemoglobin in urine. It was previously shown that the levels of urinary bladder tumor antigen (BTA) were highly correlated with urinary hemoglobin levels, indicating blood as a source of BTA in urine [26]. To exclude this possibility we confirmed the immune reaction of rh APE1/Ref-1 in hematuria samples, reconstituted by mixing urine and blood. The results, obtained using serially diluted rh APE1/Ref-1 in simulated hematuria samples, were almost completely implemented regardless of hemoglobin. Accurate quantitation of hemoglobin in hematuria may provide valuable insight into the clinical utility of APE1/Ref-1 as a BCa biomarker.

In conclusion, the overexpression of APE1/Ref-1 in BCa and its detection in blood [10] and urine solidify the potential for APE1/Ref-1 to serve as a BCa biomarker. The level of APE1/Ref-1 in urine as well as blood may be useful for identifying patients with BCa. Additionally, testing of urine APE1/Ref-1 is a convenient and noninvasive method that can distinguish BCa and thus reduce unnecessary and painful cystoscopy. Additional prospective cohort investigation using larger pools of samples, including samples from cases with various prognoses, is required to validate APE1/Ref-1 as a biomarker in the treatment of BCa.

## Figures and Tables

**Table 1:** Clinicopathological characteristics of patients with bladder cancer.

	Control	Bladder cancer		Total
		NMIBC	MIBC	
*N*	108	157	12	169
Age (mean ± SEM)	60.9 ± 16.3	68.4 ± 10.4	66.0 ± 9.3	68.23
Sex *N* (%)				
Male	62 (57.4)	133 (84.7)	10 (83.3)	205 (74.0)
Female	46 (42.6)	24 (15.3)	2 (16.7)	72 (26.0)
Smoking history	58 (48.3)	98 (62.4)	8 (66.7)	106 (62.7)
Median tumor size (cm)	n.a.	1.7	3.7	2.4
Number of tumor multiplicities	n.a.	86 (55.8)	10 (66.7)	96 (56.8)
Tumor stage	n.a.			
Ta		108 (68.8)		108 (63.9)
T1		49 (31.2)		49 (17.7)
T2			10 (83.3)	10 (3.6)
T3-4			2 (16.7)	2 (0.7)
Tumor grade	n.a.			
Low		98 (62.4)	0 (0.0)	98 (58.0)
High		59 (37.6)	12 (100.0)	71 (42.0)
No recurrence	n.a.	90 (57.3)	8 (66.7)	98 (58.0)
Previous recurrence		67 (42.7)	4 (33.3)	71 (42.0)

n.a.: nonapplicable; NMIBC: non-muscle-invasive bladder cancer; MIBC: muscle-invasive bladder cancer. Numbers in parentheses are percentages.

**Table 2:** Receiver operating curve analysis of APE1/Ref-1 values in patients with bladder cancer.

Cut-off (ng/100 *μ*L)	Sensitivity (%)	Specificity (%)	PPV (%)	NPV (%)
.2820	84.0	75.9	84.5	75.2
.2900	83.4	75.9	84.4	74.5
.2970	82.8	75.9	84.3	73.9
.3085	82.2	75.9	84.2	73.1
.3195	81.7	75.9	84.7	72.6
.3230	81.7	76.9	84.7	72.8
.3430	81.7	77.8	85.2	73.0
.3650	81.7	78.7	85.7	73.3
.3765	81.7	79.6	86.3	73.5
.3855	81.1	79.6	86.2	72.9
.3895	80.5	79.6	86.1	72.3
.3935	80.5	80.6	86.6	72.5
.3955	79.9	80.6	86.5	71.9
.3975	79.3	80.6	86.5	71.3
.4005	78.7	80.6	86.4	70.7
.4040	78.1	80.6	86.3	70.2
.4095	78.1	81.5	86.8	70.4
.4145	78.1	82.4	87.4	70.6
.4200	78.1	83.3	88.0	70.9

PPV: positive predictive value; NPV: negative predictive value.
